# Piperine and Tabersonine, but Not Lupinine, Inhibit *S. proteamaculans* Invasion of M-HeLa Cells

**DOI:** 10.3390/ijms262311320

**Published:** 2025-11-23

**Authors:** Ekaterina Bozhokina, Yuliya Berson, Olga Tsaplina

**Affiliations:** Institute of Cytology, Russian Academy of Sciences, Tikhoretsky Av. 4, St. Petersburg 194064, Russia; dr.bozhokina@mail.ru (E.B.); juletschka.ber@gmail.com (Y.B.)

**Keywords:** bacterial invasion, alkaloid, lupinine, tabersonine, piperine

## Abstract

The pathogenesis of a bacterial infection is a multistep process typically involving bacterial attachment to the host, toxin production, and/or invasion, followed by inflammation. For many bacteria, the invasion of human cells is a key step in the spread of infection and evasion of host immunity. Fusion of host cell membranes during bacterial penetration is a necessary step in invasion. The aim of this study was to evaluate the plant alkaloids, such as piperine, tabersonine, and lupinine, which have the potential to inhibit membrane fusion, fighting bacterial infection. Despite previous data on lupinine’s inhibition of membrane fusion, it has no effect on invasion or on the synthesis of the proinflammatory cytokines. This was likely due to the synthesis of the surface protein OmpX, which was a virulence factor for *S. proteamaculans* and can neutralize the effect of lupinine on the host cell membrane. Piperine and tabersonine inhibit invasion and proinflammatory cytokine synthesis in response to bacterial infection. However, tabersonine is toxic to eukaryotic cells. This makes it necessary to select an optimal concentration of tabersonine that suppresses invasion but is nontoxic to human cells. Therefore, piperine holds the greatest prospects for clinical use: it inhibits bacterial invasion while remaining nontoxic to human cells even at higher concentrations.

## 1. Introduction

Failure to follow antibiotic regimens or overuse of antibiotics has led to the emergence of antibiotic-resistant microorganisms. Antibiotic resistance is growing at an alarming rate and has become a major public health concern worldwide due to a number of reasons, including limited availability of antibiotics. Low profitability and the risk of resistance development due to long time-to-market have led to reduced investment in antimicrobial drug development [1]. The narrow spectrum of activity could preclude the use of drugs in clinical practice. Until real-time diagnostics become available and widely used in medicine, efforts should focus on finding drugs that target virulence factors specific to different bacterial species.

Currently, small-molecule drugs remain a vital component of the treatment and prevention of infections. There are two approaches to their development: the creation of drugs with direct antimicrobial activity and substances that enhance the effect of antibiotics [2]. A third, as yet clinically unproven strategy is the development of drugs that disrupt bacterial pathogenesis by inhibiting adhesins, autoinducers, and other bacterial virulence factors. The problem with antimicrobials is that they do not kill bacteria. Therefore, they cannot be used in a patient with weakened immunity. This problem should be addressed by preventing colonization and adhesion rather than blocking the action of toxins.

Bacterial pathogenesis is a multi-stage process typically involving bacterial attachment to host cells, toxin production, and/or invasion and inflammation [3]. For many bacteria, a penetration of the host cells (invasion) is a key step for spreading infection throughout the body and evading host immunity. Fusion of host cell membranes during bacterial ingestion is a key step in the invasion process [3]. Therefore, blocking this step may be considered as a potential therapeutic strategy.

This work aims to evaluate the possibility of using substances that inhibit membrane fusion as antibacterial drugs. Alkaloids isolated from plants are considered particularly promising. The use of medicinal plants in the development of new antibacterial drugs offers several advantages over synthetic or semi-synthetic antibiotics [4]. It is cost-effective and safe, both in terms of environmental impact and the minimization of adverse effects on humans. The complementary and synergistic mechanisms of plant molecule action also contribute to their potential as effective antimicrobials. Previously, the molecules lupinine, tabersonine, and piperine were discovered to inhibit the model membrane fusion [5]. Therefore, these plant alkaloids were chosen to evaluate their potential as antibacterial drugs.

The aim of this study was to evaluate the ability to fight bacterial infection using plant alkaloids. The interaction of *Serratia proteamaculans* with human cells was chosen as a model system. Gram-negative *Serratia* sp. are broad-spectrum antibiotic-resistant opportunistic pathogens in immunocompromised hosts [6]. Resistant to standard disinfection methods, these bacteria are often isolated from the respiratory and urinary tracts of patients [7]. We have previously shown that opportunistic bacteria *S. proteamaculans* were capable of penetrating into eukaryotic cells [8].

In this study, we assessed the direct effect of plant alkaloids on the growth and biofilm formation of *S. proteamaculans*. The potential of using plant alkaloids to increase antibiotic sensitivity was then assessed for a set of antibiotics to which these bacteria were sensitive. For all alkaloids, a concentration that was not toxic to human cells was determined. During the final stage, the effect of alkaloids on the intensity of bacterial invasion into eukaryotic cells and the synthesis of proinflammatory cytokines was determined. Thus, the potential for using plant alkaloids as antibacterial agents was assessed.

## 2. Results and Discussion

### 2.1. Effect of Alkaloids on Bacterial Growth and Biofilm Formation

To inhibit invasion, plant alkaloids must prevent membrane fusion. Piperine and lupinine inhibit membrane fusion at a concentration of 400 μM, while tabersonine inhibits membrane fusion at a concentration of 40 μM [5]. These concentrations were chosen as working ones. First, the direct effect of alkaloids on *S. proteamaculans* growth was assessed (Supplementary Materials). The bacteria were grown for 24 h in the presence of alkaloids. The optical density of the solution, which is proportional to the number of bacteria in the suspension, was then measured. The lupinine presence in the medium has been shown to have no significant effect on bacterial growth. Piperine and tabersonine reduced the optical density of the medium by up to 20%, regardless of the alkaloid concentration in the medium. To investigate the cause of the decrease in optical density, bacteria were grown in aerated test tubes. In the presence of tabersonine and piperine, the bacteria formed aggregates at the bottom. When these aggregates were broken up by pipetting and plated, it was shown that piperine and tabersonine in the medium at working concentrations did not affect bacterial growth.

Biofilm formation plays an important role in bacterial drug resistance [9]. A biofilm is an accumulation of microorganisms that are often stabilized by self-secreted extracellular polymeric substances consisting of polysaccharides, proteins, lipids, etc. [10]. Bacterial biofilm allows bacteria to spread infection by providing resistance to a broad spectrum of antibiotics and/or activating virulence factors [9]. Exposure to piperine increases the accumulation of reactive oxygen species, which leads to inhibition of biofilm formation by both gram-negative and gram-positive bacteria [11,12,13]. The effect of the selected alkaloids on the number of bacteria forming biofilms on the surface of the plate was assessed using 0.1% crystal violet staining (Supplementary Materials). According to the obtained results, the selected alkaloids did not affect the ability of *S. proteamaculans* to form biofilms.

### 2.2. The Effect of Alkaloids on Bacterial Sensitivity to Antibiotics

**Figure 1:**
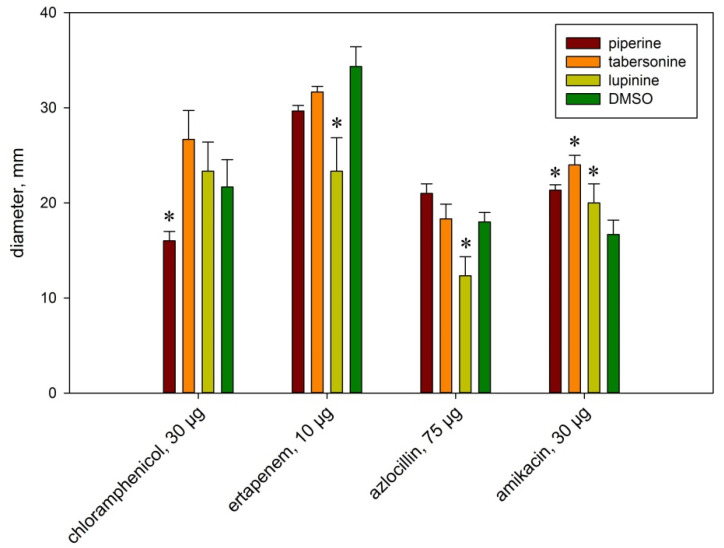
Bacterial sensitivity to antibiotics in the presence of alkaloids. Antibiotic-containing discs were placed on the agar plates with bacteria. The diameter of the inhibited bacterial growth zone around the disc was used to assess the bacterial sensitivity to the antibiotic. Experimental results represent the average of analyses performed in three replicates and independently repeated four times. Values were expressed as mean S.D. (error bars). A difference to the control was considered significant at the * *p* < 0.05 level.

Alkaloids are considered a possible drug for increasing the sensitivity of bacteria to antibiotics. It is known that piperine can be used in combination with antibiotics to increase the sensitivity of bacteria to them [14,15]. Piperine’s ability to enhance antimicrobial activity by binding other drugs is due to its involvement in inhibiting transmembrane drug efflux pumps [16]. This inhibition affects the metabolism, absorption, distribution, and excretion of substrates, leading to increased drug accumulation in bacteria [17]. The effect of piperine, lupinine, and tabersonine on the resistance of *S. proteamaculans* to antibiotics was quantitatively assessed. The possibility of regulating the sensitivity of *Serratia* to antibiotics using plant alkaloids was assessed (Figure 1). No effect of alkaloids was observed on bacterial susceptibility to aztreonam, gatifloxacin, cefepime, and meropenem. Piperine and lupinine increase the sensitivity of *S. proteamaculans* to amikacin by at least 20%, and tabersonine increases it by up to 60%. On the other hand, piperine has been shown to reduce bacterial sensitivity to chloramphenicol by 30%, and lupinine to reduce sensitivity to azlocillin and ertapenem. Thus, the use of plant alkaloids can both decrease and increase the sensitivity of bacteria to antibiotics. And when using them together, this must be taken into account.

### 2.3. Cytotoxic Effect of Alkaloids on Human Cells

To assess the intensity of invasion into human cells, bacteria are incubated with cultured cells. We have previously shown that *Serratia* efficiently penetrates M-HeLa cells [18]. Piperine was shown to effectively suppress cell viability and induce apoptosis in HRT-18 human rectal adenocarcinoma cells, DLD-1 and HT-29 colorectal adenocarcinoma cells, SW480 and Caco-2 colon adenocarcinoma cells, and SNU-16 gastric cancer cells [19,20,21,22,23]. Tabersonine, meanwhile, significantly suppresses cell viability and proliferation, inducing apoptosis in HepG2 liver cancer cells [24].

**Figure 2:**
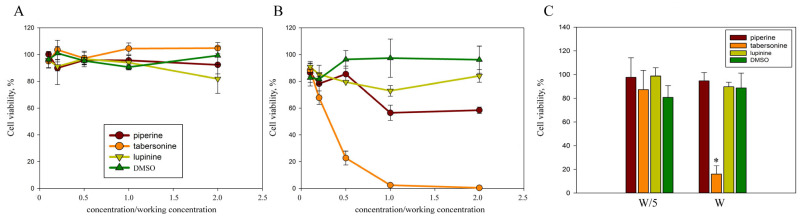
The cytotoxicity of alkaloids on human cells. M-HeLa cell viability was assessed after 2 h (**A**) and 24 h (**B**) incubation with alkaloids at concentrations ranging from twice the working concentration (400 μM piperine, 400 μM lupinine, and 40 μM tabersonine) to a tenfold dilution. (**C**) DF-2 cell viability was assessed after 24 h incubation with alkaloids at the working concentration (W) and at a fivefold dilution (W/5). Cell viability was estimated as a percentage, taking the cell viability in the control samples as 100%. Experimental results represent the average of analyses performed in three replicates and independently repeated three times. Values were expressed as mean S.D. (error bars). A difference to the control was considered significant at the * *p* < 0.05 level.

The cytotoxic effect of alkaloids on M-HeLa cells was assessed using the MTT colorimetric assay (Figure 2). During the standard time for assessing invasion intensity (2 h), all three alkaloids did not result in a cytotoxic effect on human cells (Figure 2A). After 24 h of cultivation in the presence of 400 μM piperine and 400 μM lupinine, cell viability decreased by no more than 30%, even with a two-fold increase in concentration (Figure 2B). However, 24 h of cultivation in the presence of 40 μM tabersonine led to the death of the entire cell population; in the presence of 20 μM, ¾ of the population died, and in the presence of 8 μM, no more than ¼ of the cell population died. To determine the IC50, cell survival in the presence of tabersonine was assessed in 5 μM increments. Tabersonine was shown to kill half the cell population within 24 h at a concentration of 15 μM. Thus, for further work with cell cultures, piperine and lupinine were used at a concentration of 400 μM, and tabersonine at concentrations of 40 μM and 8 μM.

Tabersonine significantly inhibited cell viability and proliferation, inducing apoptosis in HepG2 liver cancer cells in vitro and in vivo [24]. Therefore, we assessed the cytotoxicity of plant alkaloids on skin dermal fibroblasts (Figure 2C). Lupinine, piperine, and 8 μM tabersonine were shown to be nontoxic to cells, while 40 μM tabersonine killed more than 80% of DF-2 cells in culture. Thus, the cytotoxicity of tabersonine on cells was not associated with the tumor origin of the cell line.

### 2.4. Inflammatory Response to Infection in the Presence of Alkaloids

**Figure 3:**
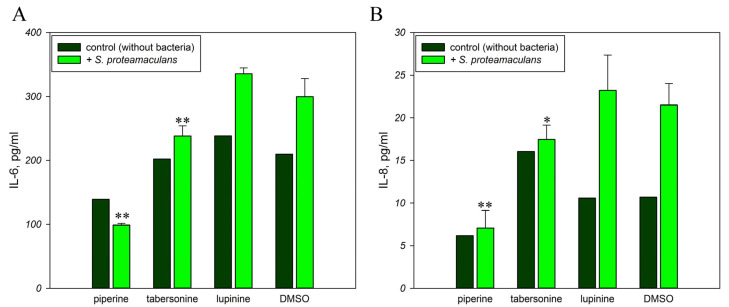
Effect of alkaloids on the inflammatory response. Secretion of interleukins IL-6 (**A**) and IL-8 (**B**) by M-HeLa cells in response to infection with *S. proteamaculans* in the presence of plant alkaloids. Experimental results represent the average of analyses performed in three replicates. Values were expressed as mean S.D. (error bars). A difference to the control was considered significant at the * *p* < 0.05, ** *p* < 0.01 level.

Piperine exerts anti-inflammatory effects on *H. pylori*-induced gastritis by suppressing IL-6 expression [25]. This alkaloid blocks the induced expression of IL-6 and IL-8 [26,27]. And tabersonine inhibited LPS-mediated production of IL-6 [28]. Infection with *S. proteamaculans* bacteria increases the secretion of proinflammatory cytokines IL-6 and IL-8 by M-HeLa cells (Figure 3). Two hours of incubation with bacteria brought an increase of IL-6 and IL-8 expression by 1.5 and 2 times, respectively. We showed that lupinine did not affect interleukin synthesis by M-HeLa cells, while piperine and tabersonine prevented the increase in interleukin levels in response to infection. Moreover, the addition of piperine to the medium inhibited the IL-6 and IL-8 synthesis by control cells without bacteria by 30% and 50%, respectively.

### 2.5. The Effect of Alkaloids on the Intensity of Invasion

**Figure 4:**
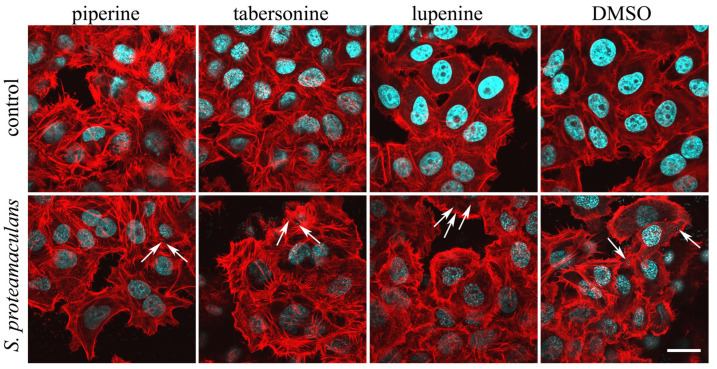
Effect of alkaloids on actin distribution in M-HeLa cells. Cells were incubated with *S. proteamaculans* bacteria for 2 h in the presence of 400 μM piperine, 40 μM tabersonine, 400 μM lupinine, and 0.1% DMSO. Control: cells were not infected with bacteria. The arrows indicate intracellular bacteria. The cytoskeleton was stained with rhodamine-phalloidin (red); bacterial and cellular DNA was stained with DAPI (blue). Scale bar: 20 µm.

To determine whether inhibition of the inflammatory response occurs due to inhibition of signaling pathways or whether tabersonine and piperine prevent invasion, we assessed the effect of alkaloids on the ability of bacteria to penetrate eukaryotic cells (Figure 4). Using confocal microscopy, we assessed the effect of alkaloids on the actin cytoskeleton of M-HeLa cells. Tabersonine and piperine, but not lupinine, induced stress fiber formation in eukaryotic cells. The addition of bacteria did not significantly reduce the number of stress fibers. However, actin cytoskeletal rearrangements are necessary for bacterial invasion [3]. Using confocal microscopy, intracellular bacteria were detected in the presence of all alkaloids.

**Figure 5:**
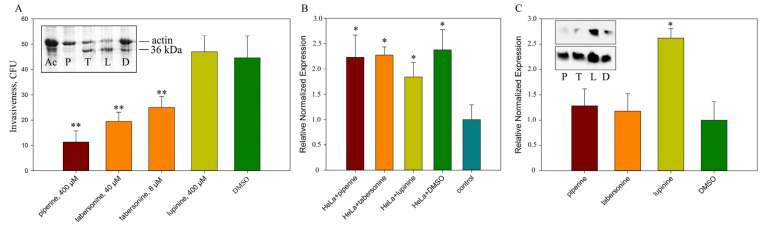
(**A**) Effect of alkaloids on the sensitivity of M-HeLa cells to bacteria. After 24 h of growth, the intensity of *S. proteamaculans* invasion into cells was assessed in the presence of alkaloids. Insert: Protealysin activity in bacterial extracts after 48 h of growth in the presence of 400 μM piperine (P), 40 μM tabersonine (T), 400 μM lupinine (L) and 0.1% DMSO (D). Control-uncleaved actin (Ac). Experimental results represent the average of analyses performed in five replicates and independently repeated three times. Values were expressed as mean S.D. (error bars). A difference to the control was considered significant at the ** *p* < 0.01 level. (**B**) The effect of contact with host cells in the presence of alkaloids on the expression of protealysin by bacteria. After 24 h of growth, *S. proteamaculans* were incubated with M-HeLa cells for 2 h in the presence of alkaloids at working concentration (400 μM piperine, 400 μM lupinine, and 40 μM tabersonine). Experimental results represent the average of analyses performed in three replicates and independently repeated three times. Values were expressed as mean S.D. (error bars). A difference to the control was considered significant at the * *p* < 0.05 level. (**C**) Effect of growth in the presence of alkaloids on the *ompX* expression by *S. proteamaculans. OmpX* gene expression by *S. proteamaculans* after 24 h of growth in the presence of 400 μM piperine (P), 40 μM tabersonine (T), 400 μM lupinine (L), and 0.1% DMSO (D). Experimental results represent the average of analyses performed in three replicates and independently repeated three times. Values were expressed as mean S.D. (error bars). A difference to the control was considered significant at the * *p* < 0.05 level. Insert: Amount of OmpX surface protein in the membranes in the same number of bacteria (determined by OD_600_) at 24 h of growth in the presence of lupinine (L), piperine (P), and tabersonine (T). Control bacteria were raised in LB broth in the presence of 0.1% DMSO (D). The results of two independent experiments are presented.

However, confocal microscopy does not allow quantitative assessment of invasion intensity. Therefore, invasion intensity was compared using quantitative microbiological methods (Figure 5A). We showed that lupinine does not affect the intensity of invasion, while piperine and tabersonine inhibited the invasion of *S. proteamaculans* into M-HeLa cells. This effect may be due to the polymerization of actin, which is necessary for invasion, when cells are treated with tabersonine and piperine.

Thus, despite the fact that all three tested alkaloids prevent membrane fusion, only piperine and tabersonine inhibit invasion by 4 and 2 times, respectively. The different invasion intensity in the presence of piperine and tabersonine may result from different effects of these alkaloids on the host cell membrane [5]. In addition, alkaloids regulate signaling pathways of eukaryotic cells involved in bacterial invasion [20,23,29,30]. Piperine causes an increase in the phosphorylation of p38 and ERK by more than 10 times but a decrease in the phosphorylation of JNK [23]. It is JNK that makes the main contribution to the invasion of *S. proteamaculans* [31]. While tabersonine significantly reduces the induction of p38 phosphorylation and, to a lesser extent, phosphorylation of ERK1/2 and JNK kinases, which are major participants in bacterial invasion [28]. Thus, differences in invasion intensity in the presence of these alkaloids may also be due to different signaling pathways that these substances inhibit.

### 2.6. Accumulation of Bacterial Virulence Factors in the Presence of Alkaloids

In addition to influencing the host cell, alkaloids can directly regulate bacterial virulence factors. The intensity of *S. proteamaculans* bacterial invasion is determined by the balance between the activity of the protease protealysin and its substrate, the bacterial surface protein OmpX [32]. Alkaloids did not affect protealysin gene expression. The activity of protealysin in bacterial extracts can be assessed by the ability to cleave actin to form a 36 kDa fragment [8]. We showed that piperine inhibits protealysin activity in extracts of bacteria grown in the presence of alkaloids (Figure 5A, inset). However, protealysin activity is detectable in bacterial extracts only after 48 h of growth. To assess the possibility of protealysin involvement in bacterial invasion at earlier stages of bacterial growth, we assessed protealysin expression during invasion. We demonstrated that contact between bacteria and eukaryotic cells leads to a two-fold increase in protealysin expression (Figure 5B). Thus, protealysin may be synthesized in response to contact with the host cell and determine differences in the intensity of bacterial invasion in the presence of alkaloids.

Proteins of the OmpX family are involved in binding to external proteins and signal transduction and are also involved in cell adhesion, invasion, and virulence [33]. The OmpX of *S. proteamaculans* binds to host cell integrins and thus increases the expression of host cell receptors involved in invasion and can trigger signaling pathways that mediate bacterial penetration [31,34]. Using RT-PCR, we demonstrated that bacterial growth in the presence of lupinine can increase *ompX* gene expression (Figure 5C). Using Western blot analysis, we demonstrated that increased expression leads to accumulation of OmpX protein anchored in the bacterial membrane (Figure 5C, insert). According to densitometric analysis, lupinine increased the amount of OmpX protein by 1.6–5.5 times (results of three independent experiments). Thus, the effect of lupinine on host cell membranes may be negated by the accumulation of the surface protein OmpX.

## 3. Materials and Methods

### 3.1. Cell Cultures, Bacterial Strains, and Growth Conditions

The cervical carcinoma M-HeLa and dermal fibroblasts of the skin DF-2 cell lines were obtained from the “Vertebrate cell culture collection” (Institute of Cytology, St. Petersburg, Russia). M-HeLa cells were grown in αMEM medium containing 1% nonessential amino acids (NEAAs) (Sigma-Aldrich, Taufkirchen, Germany) and 10% fetal bovine serum (Sigma-Aldrich, Taufkirchen, Germany). DF-2 cells were grown in DMEM/F-12 medium containing 10% fetal bovine serum (Sigma-Aldrich, Taufkirchen, Germany).

*Serratia proteamaculans* 94 was isolated as described earlier [35]. Species identification by 16S rRNA sequencing was confirmed using a Daltonik MALDI Biotyper (Bruker, Billericay, MA, USA) with a score value of 2.316 (NCBI:txid28151). *S. proteamaculans* were grown in LB medium (Sigma-Aldrich, Taufkirchen, Germany) at 30 °C with aeration. For the experiment, the bacteria were grown to the stationary growth phase, and the number of bacteria was determined by optical density at 600 nm. Aliquots containing an equal number of bacteria were taken from each bacterial suspension.

Alkaloids were used at the following working concentrations: 400 μM piperine (Sigma-Aldrich, Taufkirchen, Germany), 400 μM lupinine (Solarbio, Beijing, China), and 40 μM tabersonine (Solarbio, Beijing, China). A 1000-fold stock solution was dissolved in DMSO and stored at −20 °C. As a control, 0.1% DMSO was added to the cells.

### 3.2. Biofilm Formation

Bacteria were grown in LB for 24 h. The suspension was diluted 500 times, and 100 µL of the alkaloid-containing suspension was added to each well of a 96-well plate. Bacteria were grown in LB in a 96-well plate at 30 °C with aeration for 24 h. The number of bacteria was determined by optical density at 600 nm. The bacterial suspension was removed, and after washing with PBS, 150 µL of a 0.1% aqueous crystal violet solution was added to each well for 30 min at 37 °C to stain the formed biofilms. After removing the crystal violet solution and double washing with water, the dye was extracted from the biofilms with 150 µL of 96% ethanol for 1 h at room temperature. Optical density in control and test samples was measured spectrophotometrically at λ = 600 nm. Each measurement was carried out in triplicate, and the measurement value obtained as a result of staining the control well with LB was subtracted from the obtained data.

### 3.3. Cell Viability Assessment Using MTT Assay

We followed the 3-(4,5-dimethylthiazol-2-yl)-2,5-diphenyltetrazolium bromide (MTT) assay to assess the viability of M-HeLa and DF-2 cells [36]. In this assay, formation of blue formazan by mitochondrial activity was used to monitor the metabolic activity of M-HeLa and DF-2 cells and to determine the cell viability. MTT (3-(4,5-dimethylthiazol-2-yl)-2,5-diphenyl tetrazolium bromide) (Sigma Aldrich, Taufkirchen, Germany) was dissolved in PBS at 5 mg/mL and filtered to sterilize and remove a small amount of insoluble residue present in some batches of MTT. To estimate the cells’ viability, M-HeLa and DF-2 were seeded onto 96-well plates (5000/well) and cultured in culture medium containing 10% FBS to near confluence. Then cells were cultivated with FBS-free culture medium for 24 h and treated with different concentrations of alkaloids. Next, 7 µL MTT solution was added to each well and incubated for 2 h at 37 °C. Then, the medium was removed, and 150 µL DMSO was added to each well. After shaking for 5 min, the absorbance was detected using a microplate reader (ThermoFisher, Waltham, MA, USA) at 570 nm. Cell viability is estimated by subtracting the optical density (OD) of SDS-treated cells from the optical density (OD) of the cell samples. Cell viability was estimated as a percentage, taking the cell viability in the control samples as 100%.

### 3.4. Antibiotic Resistance

To determine antibiotic susceptibility, bacteria were grown in LB medium in the presence of 400 μM piperine, 40 μM tabersonine, 400 μM lupinine, and 0.1% DMSO for 24 h. One hundred microliters of the suspension were plated on LB agar and incubated for 1 h at room temperature until completely dry. Three to four antibiotic-containing discs (NICF, St. Petersburg, Russia) were placed in the dried Petri dishes containing bacterial cultures, spaced 2–2.5 cm from the center of the dish. The dishes containing *S. proteamaculans* were incubated at 30 °C for 24 h. Diffusion of the antibiotic into agar resulted in the formation of a growth inhibition zone around the disc, the diameter of which determines the bacterial sensitivity to the antibiotic. We analyzed the sensitivity of *S. proteamaculans* to antibiotics and determined the spectrum of antibiotics that inhibit *S. proteamaculans* growth: azlocillin (75 μg/disc), aztreonam (30 μg/disc), amikacin (30 μg/disc), gatifloxacin (5 μg/disc), chloramphenicol (30 μg/disc), meropenem (10 μg/disc), cefepime (30 μg/disc), ertapenem (10 μg/disc).

### 3.5. Cytokine Enzyme-Linked Immunosorbent Assay

Interleukin-6 (IL-6) and interleukin-8 (IL-8) concentrations were determined using sandwich-type ELISA kits A-8768 and A-8762 (Vector-best, Novosibirsk, Russia) in accordance with the manufacturer’s instructions. Briefly, 100 µL of calibration solutions, control sample, and test samples were added to the wells of the plate in duplicate. The plate was incubated for 1 or 2 h on a shaker at 37 °C, 450 rpm. Then the plates were washed, 100 µL of conjugate was added, and they were incubated for 1 h at 37 °C, 450 rpm. After washing, 100 µL of 3,3′,5,5′-tetramethylbenzidine was added, and the plate was incubated in the dark for 15 min at room temperature. The reaction was stopped by adding a stop reagent. The results were recorded spectrophotometrically using a CLARIOstar microplate reader (BMG Labtech, Ortenberg, Germany) at 450 nm wavelength. Then, a calibration curve was constructed of the dependence of optical density on the concentration of IL-6 or IL-8 in calibration solutions. The following concentrations of calibration solutions were used: for the determination of IL-8, 250, 100, 40, 15, 5, and 0 pg/mL; for the determination of IL-6, 300, 150, 50, 16.7, 5.6, and 0 pg/mL. Sensitivity metrics (LOD and LOQ): 2.0 pg/mL and 250 pg/mL for IL-8; 0.5 pg/mL and 300 pg/mL for IL-6. To assess the effect of alkaloids on the measurement, DMEM medium containing a 2-fold working concentration of alkaloids was mixed with a control sample at a 1:1 ratio. The deviation from the control sample without added alkaloids ranged from 2 to 5% and from 2 to 7% for IL-8 and IL-6, respectively. Thus, alkaloids do not affect the antibody-antigen binding process. Samples were added in 3 replicates without dilution and in 3 replicates with a 5-fold dilution. The resulting curve was used to calculate the IL-6 or IL-8 content in the test samples (pg/mL).

### 3.6. Fluorescence Microscopy

Cells were grown at 37 °C in an atmosphere of 5% CO_2_ on coverslips until a 70–80% monolayer was formed. Bacteria *S. proteamaculans* were grown in LB medium (Sigma-Aldrich, Taufkirchen, Germany) at 30 °C with aeration for 22–24 h. The bacterial suspension was centrifuged at 9600× *g*, 8 min. The pellet was resuspended in DMEM medium and added to eukaryotic cells at a ratio of 10,000 bacteria per cell. Bacteria were deposited on the surface of the host cell by centrifugation for 5 min at 2000 rpm. The host cells and bacteria were co-cultivated at 37 °C in 5% CO_2_ for 2 h. Cells were washed three times with PBS solution at each staining step. The preparations were fixed with 3.7% formaldehyde solution (Sigma-Aldrich, Taufkirchen, Germany) for 10 min and incubated for 5 min with 0.1% Triton X100. Cells were stained with rhodamine-phalloidin for 15 min to visualize the actin cytoskeleton and DAPI for 15 min to visualize DNA of bacteria and epithelial cells. The preparations were analyzed using an Olympus FV3000 microscope (Olympus Corporation, Tokyo, Japan) using a system of lasers with wavelengths of 405 (blue fluorescence), 488 (green fluorescence), and 561 nm (red fluorescence).

### 3.7. Quantitative Invasion Assay

Efficiency of invasion was evaluated by the quantitative invasion assay [13,14]. After 24 h of growth, bacteria were pelleted at 9600× *g* for 10 min; the pellets were resuspended in DMEM and added to a fresh portion of DMEM to host cells, forming a 50–70% monolayer at a ratio of 100 or 10,000 bacteria per cell. The intensity of *S. proteamaculans* invasion was the same with infection at 100 and 10,000 bacteria per cell [37]. After co-cultivating host cells and bacteria at 37 °C in 5% CO_2_ for 2 h unattached bacteria were washed out twice with PBS, and the infected cells were suspended in 0.25% trypsin-versene solution. The proportions of viable cells were measured after staining with trypan blue by counting in a Goryaev chamber. Cell viability in all experiments was at least 90%. To quantify the effectiveness of invasion, suspension of the infected cells was incubated in DMEM containing mixture of antibiotics (80 μg/mL kanamycin and 40 μg/mL gentamicin) for 1 h at 37 °C to kill extracellular bacteria, and then cells were lysed with 1.5% sodium deoxycholate, quickly diluted with cold LB medium, and aliquots of the resulting suspension were plated on LB-agar to determine the number of colony forming units (CFU) of intracellular bacteria. The intensity of invasion was determined as the number of CFU normalized to the number of viable eukaryotic cells determined after incubation with bacteria. The results for each experiment were the average of an assay performed in triplicate and independently repeated three times.

### 3.8. Western Blot Analysis

*Serratia proteamaculans* 94 were grown to the stationary growth phase, and the number of bacteria was determined by optical density at 600 nm. Aliquots containing an equal number of bacteria were taken from each bacterial suspension. To isolate the membrane proteins, bacteria were pelleted by centrifugation at 12,000× *g* for 10 min, the pellets were re-suspended in buffer G, and the bacteria were lysed by seven cycles of freezing and thawing and pelleted by centrifugation at 12,000× *g* for 10 min. The pellet was resuspended in 2% sodium lauryl sarcosinate (Sigma Aldrich, USA) in 10 mM PBS, incubated at room temperature for 1 h, pelleted by centrifugation at 12,000× *g* for 10 min; and resuspended in 1% sodium lauryl sarcosinate. Suspensions were centrifuged at 12,000× *g* for 10 min; the pellet was resuspended in buffer G and analyzed. The Bradford assay was used to ensure that the concentration of protein isolated from the same number of bacteria remained the same in all samples.

Equal volumes of samples were fractionated by SDS-PAGE and transferred to a Hybond ECL membrane according to the manufacturer’s instructions (GE Healthcare, Chalfont Saint Giles, UK). The membrane was incubated with 5% nonfat milk in PBS for 40 min to prevent nonspecific binding of antibodies and then incubated with rabbit antibodies against OmpX (PA5-144396) at a dilution 1:2000 (Thermo Fisher Scientific, Waltham, MA, USA) at room temperature for 1 h. The membrane was then washed three times with washing buffer (5% nonfat milk, 0.1% Tween 20, PBS) for 10 min and incubated for 2 h with the secondary antibodies (1:20,000) against rabbit IgG conjugated with horseradish peroxidase IgG (Thermo Fisher Scientific, Waltham, MA, USA). The blots were washed with washing buffer three times and developed using SuperSignal West FEMTO Chemiluminescent Substrate (Thermo Fisher Scientific, Waltham, MA, USA) according to the manufacturer’s recommendations.

### 3.9. Limited Proteolysis Assay

Rabbit skeletal muscle actin used as a substrate for protealysin was isolated by a standard procedure of Spudich and Watt [38]. G-actin in buffer G (0.2 mM ATP, 0.1 mM CaCl_2_, 5 mM Tris-HCl, pH 7.5, 0.02% NaN_3_) was stored as aliquots (1–3 mg/mL) at −20 °C for a single use. To determine the ability of bacterial extracts to cleave actin, bacteria were pelleted by centrifugation at 12,000× *g* for 10 min, the pellets were re-suspended in buffer G, and the bacteria were lysed by seven cycles of freezing and thawing. The bacterial extracts were clarified by centrifugation at 12,000× *g* for 10 min. The clarified bacterial extract was mixed with an equal volume of actin and incubated for 18 h at 4 °C. The reaction was stopped by addition of an equal volume of the electrophoresis sample buffer containing 4% SDS and 125 mM Tris-HCl, pH 6.8, followed by a 5 min boiling. The digestion products were analyzed by SDS PAGE [39]. The actinase activity was determined by the appearance of the 36 kDa actin fragment.

### 3.10. Real-Time RT-PCR

Total RNA was extracted from cells using the Dia-M Extraction Kit according to the manufacturer’s instructions (Dia-M, Moscow, Russia). Reverse transcription and amplification were performed with the BioMaster HS-qPCR SYBR Blue (Biolabmix, Novosibirsk, Russia) using the CFX96 Touch Real-Time PCR machine (Bio-Rad, Irvine, CA, USA).

The steps included initial cDNA synthesis at 45 °C for 30 min, denaturation at 95 °C for 5 min, and 40 cycles of 95 °C for 30 s, 60 °C for 30 s, and 72 °C for 30 s. Each sample was run in triplicate. Target gene expression was normalized to the expression of an S12 ribosomal protein bacterial housekeeping gene and calculated using the 2^−ΔΔCT^ method. The gene-specific primer pairs (Evrogen, Moscow, Russia) designed using BLAST-primer software (2.13.0) and used for real-time PCR are listed in Table 1.

### 3.11. Statistical Analysis

Each quantitative experiment was repeated 3–5 times, with three repetitions in each experiment. In the figures, values were expressed as the mean ± S.D. (error bars) of a representative experiment. Significance testing in comparisons is based on Student’s *t*-tests for pairs and analysis of variance (ANOVA) with Excel Data Analysis Pack. The difference was considered significant at the *p* < 0.05 level.

## 4. Conclusions

When penetrating the human body, bacteria can evade the host’s immune system by invading the cell’s cytoplasm. Invasion is the key stage in spreading many infections. Bacteria can penetrate host cells via a trigger or zipper mechanism [3]. Regardless of the mechanism of bacterial penetration, invasion requires cytoskeletal rearrangements, fusion of the host cell membrane, and the formation of a bacterium-containing vacuole. During our work, we demonstrated that all tested alkaloids can enhance the action of amikacin, which acts by creating fissures in the outer membrane of the bacterial cells [40]. Piperine and tabersonine can inhibit the invasion and synthesis of proinflammatory cytokines in response to bacterial infection by influencing the transmembrane distribution of lateral pressure. However, lupinine had no effect on either invasion or the synthesis of proinflammatory cytokines. These differences may be due to differences in their effects on both host cells and bacteria. Piperine and tabersonine, but not lupinine, induce actin polymerization and stress fiber formation, making actin less accessible. Actin polymerization in the host cell’s perimembrane cytoskeleton is required for invasion. Furthermore, lupinine, but not piperine or tabersonine, enhances the expression of the bacterial surface protein OmpX, which is a virulence factor of *S. proteamaculans* and can compensate for the effect of lupinine on the host cell membrane. Tabersonine is toxic to eukaryotic cells, which necessitates the selection of an optimal concentration of the active substance that inhibits invasion but is not yet toxic to human cells. This concentration was found in the work for the model system of host–cell interaction with *S. proteamaculans*. These results were obtained on the M-HeLa cell line model, which is not a model of the epithelial barrier and does not allow one to evaluate the pharmacokinetics (PK) and pharmacodynamics (PD) of the drugs and exclude off-target effects at higher concentrations of alkaloids. However, based on this study, it can be concluded that piperine has proven to be the most promising for clinical use; it inhibits bacterial invasion while being nontoxic to human cells even at a twofold increase in the working concentration.

## Figures and Tables

**Table 1:** Gene-specific primer pairs.

Target Gene	Primer Sequences
*ompX*	Forward 5′-GCAGTAGCAGCCTGTGTATTA-3′
	Reverse 5′-TTGGGCGTTGTCGATGTT-3′
*protealysin*	Forward 5′-GGTGAAGTCATCCGCGATATT-3′
	Reverse 5′-ATCAGCCAGTCGGCTTTATC-3′
*S12 ribosomal protein*	Forward 5′-CAGAAACGTGGCGTATGTACT-3′
	Reverse 5′-CGAGCTTGCTTACGGTCTTTA-3′

## Data Availability

The original contributions presented in this study are included in the article/Supplementary Materials. Further inquiries can be directed to the corresponding author.

## Supplementary Materials

The following supporting information can be downloaded at: https://www.mdpi.com/article/10.3390/ijms262311320/s1.
