# Supplementary material for: Piperine and Tabersonine, but Not Lupinine, Inhibit S. proteamaculans Invasion of M-HeLa Cells
Source: Int J Mol Sci. 2025 Nov 23;26(23):11320. doi: 10.3390/ijms262311320 (PMC12692479; doi:10.3390/ijms262311320)
Supplement: Supplementary file 1 [file ijms-26-11320-s001.zip › ijms-3925645-supplementary.pdf]

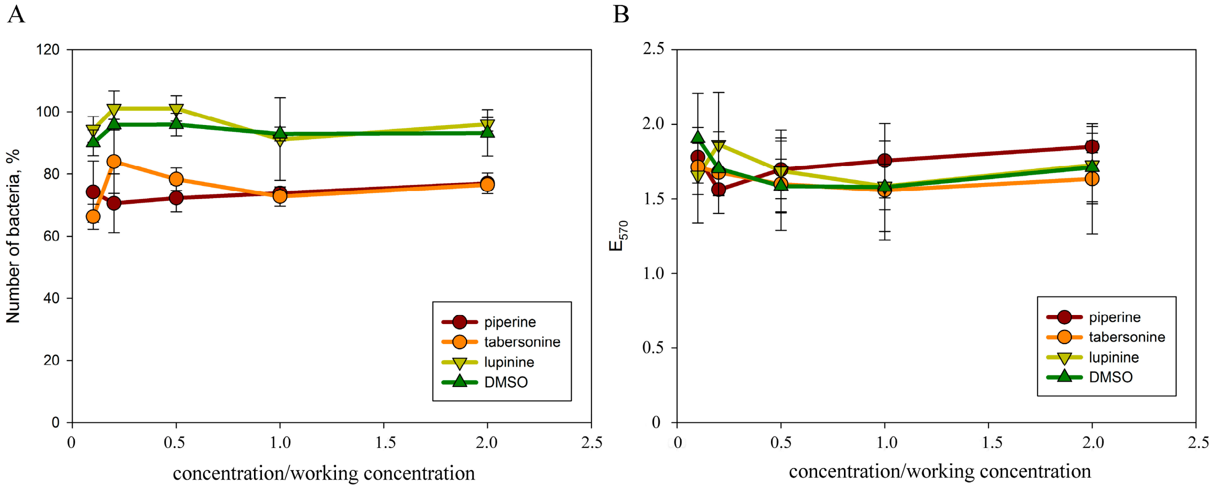

Supplementary Figure S1. Bacterial growth and biofilm formation in the presence of alkaloids. Alkaloids were added at concentrations ranging from twice the working concentration (400 µM piperine, 400 µM lupinine and 40 µM tabersonine) to a tenfold dilution. The optical density of the solution is proportional to the number of bacteria in the suspension (A) and on the surface of the culture plate (B). Experimental results represent the average of analyses performed in three replicates and independently repeated three times.
